# Unobtrusive Estimation of Cardiovascular Parameters with Limb Ballistocardiography

**DOI:** 10.3390/s19132922

**Published:** 2019-07-01

**Authors:** Yang Yao, Sungtae Shin, Azin Mousavi, Chang-Sei Kim, Lisheng Xu, Ramakrishna Mukkamala, Jin-Oh Hahn

**Affiliations:** 1 College of Medicine and Biological Information Engineering, Northeastern University, Shenyang 110169, China; 2 Department of Mechanical Engineering, University of Maryland, College Park, College Park, MD 20742, USA; 3 School of Mechanical Engineering, Chonnam National University, Gwangju 61186, Korea; 4 Department of Electrical and Computer Engineering, Michigan State University, East Lansing, MI 48824, USA

**Keywords:** ballistocardiography, ballistocardiogram, blood pressure, stroke volume, cardiac output, total peripheral resistance, photoplethysmography, photoplethysmogram

## Abstract

This study investigates the potential of the limb ballistocardiogram (BCG) for unobtrusive estimation of cardiovascular (CV) parameters. In conjunction with the reference CV parameters (including diastolic, pulse, and systolic pressures, stroke volume, cardiac output, and total peripheral resistance), an upper-limb BCG based on an accelerometer embedded in a wearable armband and a lower-limb BCG based on a strain gauge embedded in a weighing scale were instrumented simultaneously with a finger photoplethysmogram (PPG). To standardize the analysis, the more convenient yet unconventional armband BCG was transformed into the more conventional weighing scale BCG (called the synthetic weighing scale BCG) using a signal processing procedure. The characteristic features were extracted from these BCG and PPG waveforms in the form of wave-to-wave time intervals, wave amplitudes, and wave-to-wave amplitudes. Then, the relationship between the characteristic features associated with (i) the weighing scale BCG-PPG pair and (ii) the synthetic weighing scale BCG-PPG pair versus the CV parameters, was analyzed using the multivariate linear regression analysis. The results indicated that each of the CV parameters of interest may be accurately estimated by a combination of as few as two characteristic features in the upper-limb or lower-limb BCG, and also that the characteristic features recruited for the CV parameters were to a large extent relevant according to the physiological mechanism underlying the BCG.

## 1. Introduction

The ballistocardiogram (BCG) is the recording of body movement (including displacement, velocity, and acceleration) in response to the ejection of the blood by the heart. In the absence of any external force acting on the body, the center of mass of the body must remain unchanged. Hence, as the blood circulates in the body, the rest of the body moves in the opposite direction to the circulating blood so that the center of mass of the entire body is maintained. This body movement may be recorded using a wide range of BCG instruments, such as a force plate [1,2,3], weighing scale [4,5,6,7], bed [8,9], chair [10,11,12], and wearables [13,14,15]. Being a response to the circulation of the blood, the BCG may be closely associated with the cardiovascular (CV) functions and thus possess clinical value. In fact, a recent study by us elucidated that the BCG is primarily attributed to the interaction of blood pressure (BP) at the aortic inlet and outlet as well as the apex of the aortic arch [16]. Hence, the BCG waveform is largely shaped by the aortic BP waveforms and may thus serve as a window through which the shape of the aortic BP waveforms can be inferred (at least to a certain extent).

The measurement of clinically significant CV parameters often requires inconvenient instruments and even invasive procedures. For example, the gold standard arterial BP waveform is measured by invasive arterial catheterization [17]. There are non-invasive options such as volume-clamping techniques [18,19] and applanation tonometry [20], but these techniques require costly equipment and/or trained operators. The gold standard stroke volume (SV), cardiac output (CO), and total peripheral resistance (TPR) likewise require inconvenient and costly procedures such as dye injection [21], echocardiography [22], impedance cardiography [23], and electrical impedance tomography [24]. The CV parameters have also been derived indirectly using the so-called pulse contour methods [25,26,27,28,29]. These methods have been extensively investigated and demonstrated to be successful. Yet, the techniques still necessitate the measurement of (invasive or non-invasive) arterial BP waveforms.

Considering that the shape of the BCG may originate from the aortic BP waveforms, it is quite reasonable to conceive that the BCG (especially the characteristic features therein) may have a close relationship to the CV parameters. Combined with the unobtrusiveness of the BCG instrumentation, such a capability may open up unprecedented possibilities for ultra-convenient estimation of CV parameters in daily life. Regardless, the existing body of work on the use of the BCG for CV parameter estimation is quite sparse other than cuff-less BP. Indeed, it is only recently that a few pioneering studies to investigate the feasibility of the BCG for CV parameter monitoring appeared, including diastolic BP (DP) and systolic BP (SP) [1,2,14,30,31,32], SV and CO [30,33], peripheral blood oxygenation [30], and preload and afterload [34].

Motivated by this opportunity and limitations of the state-of-the-art techniques, the goal of this work was to investigate the potential of the limb BCG for unobtrusive estimation of CV parameters. The BCG in the head-to-foot direction was instrumented at the upper limb site using an accelerometer embedded in a wearable armband and at the lower limb site using a strain gauge embedded in a customized weighing scale, respectively, simultaneously with a finger photoplethysmogram (PPG). The weighing scale BCG is to a large extent analogous to the traditional whole-body BCG [16]. Thus, its physical implications may be drawn from earlier work on the BCG [35,36,37]. In contrast, there is relatively little prior work on the armband BCG, since these so-called wearable BCG has gained interest only recently by virtue of its convenience in instrumentation. Hence, the weighing scale BCG and the armband BCG may present contrasting trade-off between the accuracy of CV parameter estimation and compatibility to wearable implementation. To standardize the analysis of these distinct BCG signals, the armband BCG was transformed into the weighing scale BCG (called the synthetic weighing scale BCG) using a signal processing procedure. The characteristic features were extracted from these BCG and PPG waveforms in the form of wave-to-wave time intervals, wave amplitudes, and wave-to-wave amplitudes as well as BCG-PPG pulse transit time (PTT; the time interval between a major wave in the BCG and the diastolic foot of the PPG) [1,2,31]. Then, the relationship between the characteristic features associated with (i) the weighing scale BCG-PPG pair and (ii) the synthetic weighing scale BCG-PPG pair versus the CV parameters (including DP, pulse BP (PP), and SP, SV, CO, and TPR) was analyzed using the multivariate linear regression analysis.

## 2. Materials and Methods

**Figure 1:**
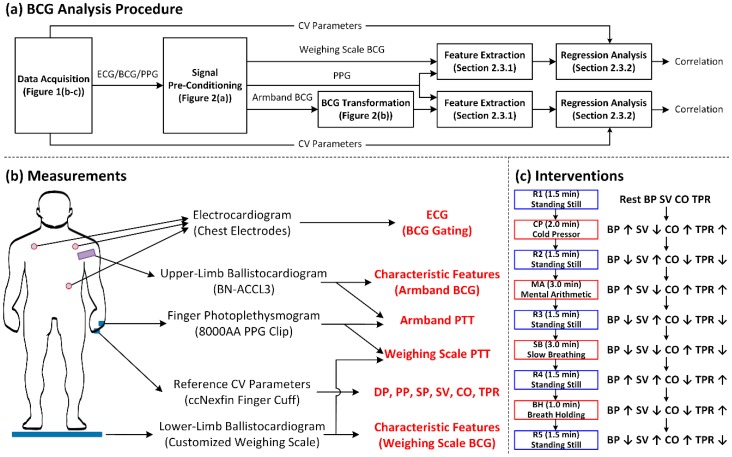
Study design and experimental protocol. (**a**) BCG signal analysis procedure to investigate the potential of limb BCG for cardiovascular (CV) parameter estimation. (**b**) Instrumented physiological signals. (**c**) Hemodynamic interventions. ECG: electrocardiogram. BCG: ballistocardiogram. PTT: pulse transit time. DP: diastolic pressure. PP: pulse pressure. SP: systolic pressure. SV: stroke volume. CO: cardiac output. TPR: total peripheral resistance.

To investigate the potential of the limb BCG for CV parameter estimation, the upper-limb and lower-limb BCG signals were analyzed with the following procedure: (i) experimental data acquisition; (ii) signal pre-conditioning; (iii) signal processing to transform the armband BCG into the weighing scale BCG; (iv) feature extraction; and (v) multivariate regression analysis (Figure 1a).

### 2.1. Experimental Protocol

Under the approval obtained from the University of Maryland Institutional Review Board (IRB) and written informed consent, human subject study was conducted in 17 young healthy volunteers (age 25 ± 5 years old; gender 12 male and 5 female; height 174 ± 10 cm; weight 74 ± 17 kg), in strict accordance with the IRB guidelines.

From each subject, a wide variety of physiological signal waveforms required for investigating the relationship between the upper-limb (i.e., arm) and lower-limb (i.e., leg) BCG and the CV parameters was instrumented using off-the-shelf sensors as follows. First, the ECG was instrumented using three gel electrodes in a modified Lead II configuration interfaced to a wireless amplifier (BN-EL50, Biopac Systems, Goleta, CA, USA). Second, the reference CV parameters (including the BP waveform, SV, CO, and TPR) were instrumented using a fast servo-controlled finger cuff embedded with a blood volume waveform sensor on the ring finger of a hand to implement the volume clamping method [18,19] (ccNexfin, Edwards Lifesciences, Irvine, CA, USA). Third, the upper-limb BCG (called hereafter the armband BCG) was instrumented using a high-resolution accelerometer embedded in an armband equipped with a wireless amplifier (BN-ACCL3, Biopac Systems, Goleta, CA, USA). Fourth, the lower-limb BCG (called hereafter the weighing scale BCG) was instrumented using a strain gauge embedded in a customized weighing scale (BC534, Tanita, Tokyo, Japan). Fifth, the PPG signal was instrumented using a finger clip sensor (8000AA, Nonin Medical, Plymouth, MN, USA). All the devices were interfaced to a laptop computer by way of a data acquisition unit (MP150, Biopac Systems, Goleta, CA, USA) to synchronously instrument all the waveforms at 1 kHz sampling rate (Figure 1b).

The aforementioned physiological signal waveforms were acquired while the subjects underwent four hemodynamic interventions (Figure 1c). Each subject stood still for 1.5 min for an initial rest state (R1). Then, the subject underwent the cold pressor intervention (CP) for 2 min, in which the subject was asked to immerse a free hand in ice water. Followed by standing still for 1.5 min for a second rest state (R2), the subject underwent the mental arithmetic intervention (MA) for 3 min, in which the subject was asked to repeatedly add the digits of a three-digit number and add the sum to the original number. Followed by standing still for 1.5 min for a third rest state (R3), the subject underwent the slow breathing intervention (SB) for 3 min, in which the subject was asked to take deep and slow breaths. Followed by standing still for 1.5 min for a fourth rest state (R4), the subject underwent the breath holding intervention (BH), in which the subject was asked to hold breath after normal exhalation. Lastly, the subject stood still for 1.5 min for a fifth rest state (R5). During the study, the subjects were asked to stand on the customized weighing scale with their arms placed at the side and still, and their movements minimized. Signal acquisition was continuously made throughout these states. 

### 2.2. Signal Pre-Conditioning

**Figure 2:**
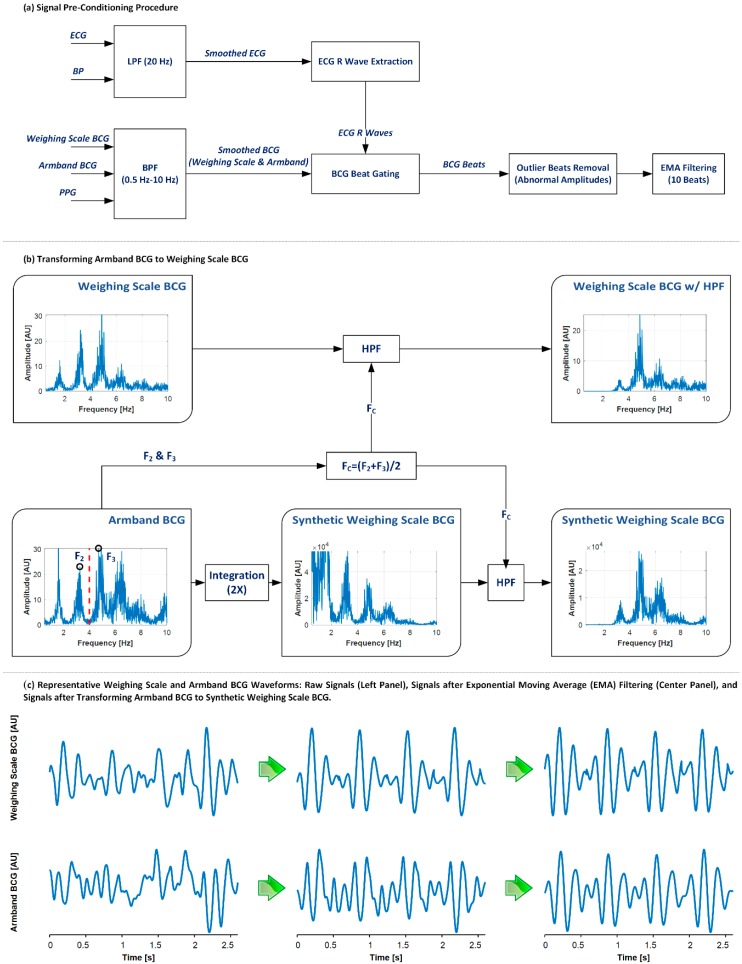
Procedure for signal pre-conditioning and transformation of armband BCG to weighing scale BCG. (**a**) Signal pre-conditioning procedure. LPF: low-pass filtering. BPF: band-pass filtering. EMA: exponential moving average. (**b**) Procedure for transforming armband BCG to weighing scale BCG. F_2_, F_3_, F_C_: frequencies associated with 2nd (F_2_) and 3rd (F_3_) spectral peaks and the band (marked as red vertical line in the armband BCG signal) of high-pass filtering (HPF). (**c**) Representative weighing scale and armband BCG waveforms: raw signals (left), signals after EMA filtering (center), and signals after transforming the armband BCG to the synthetic weighing scale BCG.

In each subject, the acquired data were segmented into nine periods: R1, CP, R2, MA, R3, SB, R4, BH, and R5. Then, the physiological signal waveforms were pre-conditioned as follows on a period-by-period basis. First, the signals were smoothed via zero-phase filtering: the ECG and BP by a 1st-order Butterworth low-pass filter with a cut-off frequency of 20 Hz, and the BCG and PPG by a 2nd-order Butterworth band-pass filter with a pass band of 0.5~10 Hz. Second, the ECG R wave was extracted using the Pan Tompkins method. Third, the BCG and PPG beats were gated with the time instants corresponding to 10% of cardiac period before the R wave as gating locations. Fourth, beats associated with the low-quality armband and/or weighing scale BCG waveforms were discarded, by (i) calculating the amplitudes associated with all the armband and weighing scale BCG beats, and (ii) removing the beats associated with extraordinarily large or small BCG amplitude (i.e., outside of 3 scaled median absolute deviations (with the scaling factor of 1.4826) around the median amplitude) [38]. Fifth, the armband and weighing scale BCG signals were smoothed using a 10-beat exponential moving average filter to suppress the adverse impact of motion artifacts. The signal pre-conditioning procedure is depicted in Figure 2a.

### 2.3. Analysis of Weighing Scale Ballistocardiogram (BCG) for Cardiovascular (CV) Parameter Estimation

The weighing scale BCG was analyzed to investigate its association with the CV parameters in the following steps: (i) feature extraction and (ii) multivariate regression analysis.

#### 2.3.1. Feature Extraction

The weighing scale BCG was labeled for the major I, J, and K waves as follows. The J wave was determined by finding the maximum peak in each BCG beat appearing after the ECG R wave. Then, the I and K waves were determined by finding the local minima right before and after the J wave, respectively. The foot of the PPG was determined using the intersecting tangent method [39]. By using these labels, a total of 16 characteristic features listed in Table 1 was constructed.

The reference CV parameters were computed as follows. In each cardiac beat, diastolic (DP) and systolic (SP) BP were computed as the minimum and maximum values in the BP waveform, while pulse pressure (PP) was computed as the difference between DP and SP. SV, CO, and TPR were computed as the mean values of the recorded SV, CO, and TPR values in each cardiac beat.

#### 2.3.2. Data Analysis

The data were analyzed in the following steps. First, the outliers in the extracted characteristic features were identified and removed. Second, the sample size of the characteristic features was increased. Third, the relationship between the characteristic features and the CV parameters were analyzed. The analysis was performed on the subject-by-subject basis.

First, the outliers in the characteristic features were extracted from the BCG and PPG signals as follows. In each of the nine rest and intervention periods associated with each subject, we examined the time series sequences of the characteristic features. Each 3 consecutive samples in the time series were inspected for possible outliers in a 9-sample window (including 3 samples before and 3 samples after the inspected samples). An outlier was identified if a sample was outside of 3 scaled median absolute deviations around the median of the 9 characteristic feature samples. If >75% of the beats in a period were removed, the period itself was excluded from subsequent analysis. Subjects in which <6 rest and intervention periods are available for analysis was also excluded from subsequent analysis.

Second, we increased the sample size of the characteristic features using the bootstrap technique similar to prior work [40,41] so as to conduct robust regression analysis (i.e., to reliably determine the coefficients in the regression models). More specifically, in each of the nine rest and intervention periods associated with each subject, the time intervals at which the CV parameters and the characteristic features attained stable extrema were determined (see Table 2 for the definition of the extrema). Then, 11 samples in the vicinity of the extrema were taken, the average of which were used as the representative CV parameter and characteristic feature values associated with the period. In addition, each of the CV parameters and characteristic features were approximated as the corresponding parametric bootstrap based on the mean and standard deviation of the 11 samples. Then, 100 bootstrap samples were created using the Monte Carlo method. Each bootstrap sample was created by (i) creating 11 random Monte Carlo samples and (ii) taking their average. Hence, up to 900 bootstrap samples (corresponding to the nine rest and intervention periods) were created in each subject. In each subject, the bootstrap samples of the CV parameters and characteristics features associated with all the rest and intervention periods were merged for multivariate regression analysis.

Third, multivariate linear regression analysis was conducted at the individual subject level to investigate the potential of the weighing scale BCG for unobtrusive estimation of CV parameters. First, multivariate linear regression models associated with each of the CV parameters were developed using the bootstrap samples. Then, the validity of these models was tested using the representative CV parameters and characteristic features at the extrema associated with all the available rest and intervention periods of the subject (≤9; Figure 1c). The goal of the multivariate regression analysis was to determine (i) the most predictive characteristic features for the CV parameters as well as (ii) the number of characteristic features required to achieve high degree of correlation (r ≥ 0.7) with the CV parameters for accurate estimation. Hence, we considered all possible combinations of the characteristics features exhaustively, and selected the models exhibiting high degree of correlation and equipped with physiologically relevant characteristic features (e.g., as suggested by our prior work [16,42]). The Pearson’s correlation coefficient was used for determining the univariate characteristics features closely correlated with the CV parameters as well as for assessing the performance of the multivariate linear regression models.

### 2.4. Analysis of Armband BCG for CV Parameter Estimation

The armband BCG was analyzed to investigate its association with the CV parameters in the following steps: (i) transformation of the armband BCG to the weighing scale BCG, (ii) feature extraction, and (iii) multivariate regression analysis.

#### 2.4.1. Transformation of Armband BCG to Weighing Scale BCG

The armband BCG and the weighing scale BCG are distinct in waveform morphology due to the difference in the measurement modality involved: the former is an acceleration measurement whereas the latter is a displacement measurement. Our prior work on the physical mechanisms and implications of the BCG [16,42] suggests that the relationship between the upper-limb acceleration BCG and CV parameters is obscure due to the mechanical body filtering effect compared with the lower-limb displacement BCG. Hence, the armband BCG was transformed into an equivalent weighing scale BCG. Given that the primary source of the discrepancy between the armband BCG and the weighing scale BCG is the measurement modality (i.e., accelerometer versus strain gauge) if the body is assumed to be rigid, this was accomplished by applying two integrations to the armband BCG (Figure 2b). More specifically, the armband BCG was integrated in time twice using the trapezoidal method to yield the synthetic weighing scale BCG. Then, the synthetic weighing scale BCG was zero-phase filtered using a 4th-order Butterworth high-pass filter to remove the low-frequency drift therein. The cut-off frequency of the filter was determined so that the power spectra (especially in terms of the primary spectral peaks) associated with the weighing scale BCG and the synthetic weighing scale BCG were made consistent. The comparison of the power spectra associated with the weighing scale BCG and the synthetic weighing scale BCG showed that the latter exhibited largely higher spectra up to the 2nd spectral peak compared to the former (Figure 2b). Hence, the cut-off frequency was determined empirically as the average of the 2nd and 3rd peaks in the BCG power spectrum. Practically, the cut-off frequency can be computed easily from the heart rate as 2.5 times the heart rate, since the spectral peaks in the BCG represent the heart rate and its harmonics. The above-described procedure was performed in each subject on a period-by-period basis.

We quantitatively assessed the beat-by-beat quality of the weighing scale BCG and the synthetic weighing BCG calculated from the armband BCG via the following criteria: (1) $\frac{\left\|s\left[i\right]-\bar{s}\right\|}{\left\|\bar{s}-m\right\|}>1$, where s[i] is individual BCG beat in a (rest or intervention) period, $\bar{s}$ is the ensemble average of all beats in the period, m is the mean of $\bar{s}$; (2) correlation coefficient between s[i] and $\bar{s}$ less than 0.5 in each period; (3) a peak with a prominence [43,44] of >0.25 is detected from the 2nd derivative of the BCG waveform from I wave to K wave as a measure of distortion in the BCG waveform. All beats not fulfilling these criteria were removed from further analysis.

#### 2.4.2. Feature Extraction and Data Analysis

Feature extraction and data analysis were conducted in the same way as the weighing scale BCG, as described in detail in Section 2.3.

## 3. Results

### 3.1. Experimental Data

**Figure 3:**
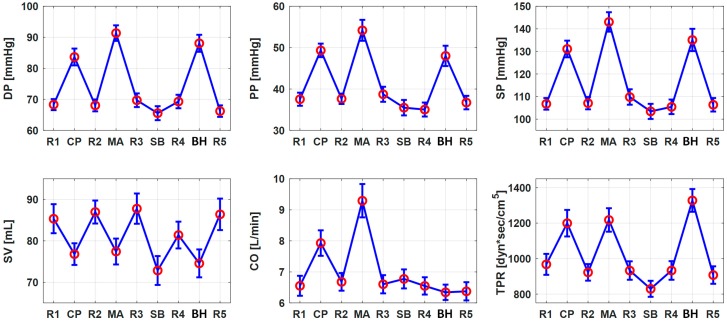
Group-average changes in the cardiovascular parameters in response to hemodynamic interventions (mean ± standard error (SE)).

Figure 3 shows the trends of the changes in the CV parameters in response to the hemodynamic interventions employed in this work. DP, PP, SP, and TPR increased in response to CP, MA, and BH, while it decreased in response to SB. Likewise, CO increased in response to CP and MA. But, it increased modestly in response to SB and decreased modestly in response to BH. SV decreased in response to all the hemodynamic interventions. Noting that CO increased in CP and MA, the decrease in SV may be attributed to a large increase in heart rate which shortens the left ventricular ejection time yet still increases CO [45]. On the other hand, the decrease in SV in SB and BH may be associated with the marginal change in CO and decrease in heart rate, which is consistent with the findings of prior studies [46,47,48]. These trends were used in defining the extrema associated with the CV parameters in Table 2.

### 3.2. CV Parameter Estimation with Weighing Scale BCG

**Figure 4:**
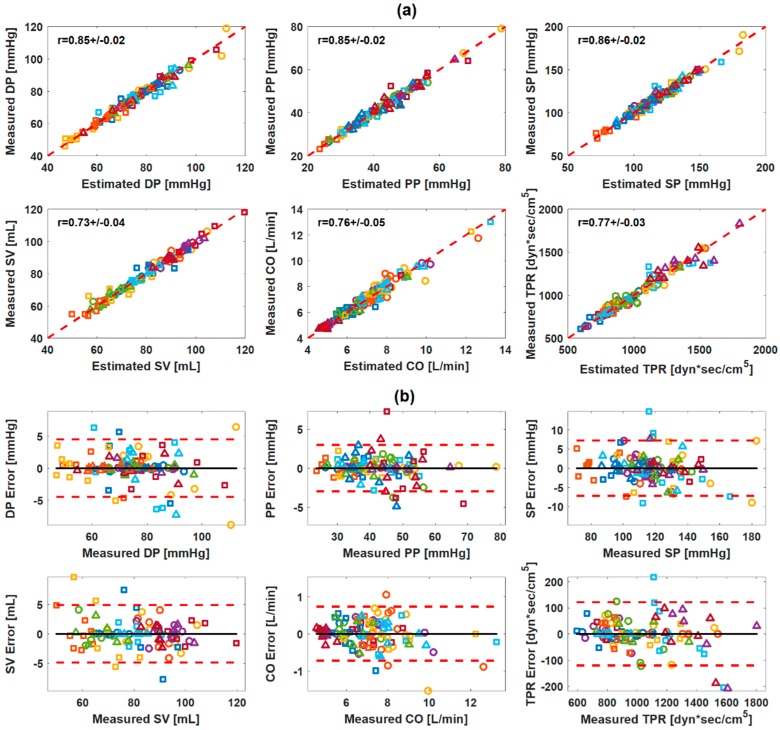
Correlation and Bland–Altman plots between measured versus regressed cardiovascular parameters: weighing scale ballistocardiogram (BCG). (**a**) Correlation plots. (**b**) Bland–Altman plots. Black solid line: bias. Red dashed lines: confidence interval.

The number of subjects available for multivariate linear regression analysis after the outlier removal (i.e., subjects with ≥6 rest and intervention periods available for analysis; see Section 2.3 for details) was ≥14 for all the CV parameters associated with the weighing scale BCG. Multivariate linear regression analysis suggested that each of the CV parameters of interest may be accurately estimated by a combination of as few as two characteristic features. In contrast, the best correlation coefficients achieved by univariate characteristic features were on the average high for DP (0.81) and SP (0.82) but not sufficiently high for the remaining CV parameters (<0.65). For the weighing scale BCG at the univariate level, DP was correlated well with PTT_I_ (r = −0.81 ± 0.02) and PTT_J_ (r = −0.69 ± 0.04), PP was correlated reasonably with PTT_I_ (r = −0.65 ± 0.05) and PTT_J_ (r = −0.57 ± 0.07) as well as A_JK_ (0.54 ± 0.07) and A_IJ_ (0.53 ± 0.07), and SP was correlated well with PTT_I_ (r = −0.82 ± 0.02) and PTT_J_ (r = −0.72 ± 0.04). SV was correlated most strongly with A_J_ (r = 0.50 ± 0.09). CO was correlated with T_JJ_ (r=-0.57 ± 0.11), and to a lesser extent, with PTT_I_ and PTT_J_. TPR was likewise correlated with PTT_I_ (r = −0.58 ± 0.07) and PTT_J_ (r = −0.52 ± 0.09) but also with T_JJ_ (r = 0.54 ± 0.07). Table 3 shows the best-performing univariate and bivariate regression models associated with the weighing scale BCG. Figure 4a shows the correlation plot and Figure 4b the Bland–Altman plot between measured versus regressed CV parameters associated with the weighing scale BCG.

### 3.3. CV Parameter Estimation with Armband BCG

The signal processing procedure (Figure 2) drastically improved the correlation between the measured versus synthetic weighing scale BCG compared to the correlation between the measured weighing scale versus armband BCG, both at all the individual rest and intervention states as well as across all the rest and intervention states (r = 0.70 versus r = 0.52 on the average).

**Figure 5:**
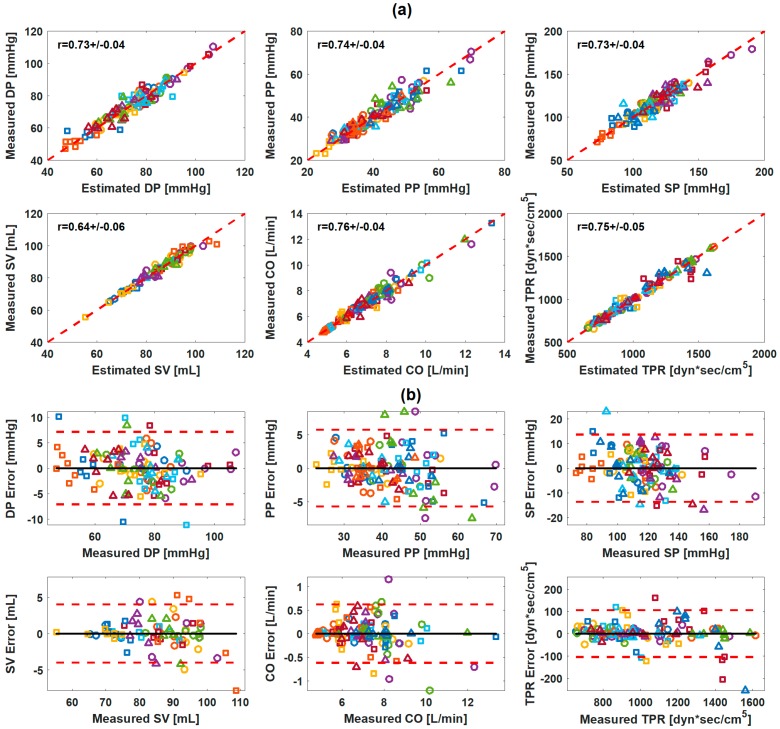
Correlation and Bland–Altman plots between measured versus regressed cardiovascular parameters: synthetic weighing scale ballistocardiogram (BCG) transformed from armband BCG. (**a**) Correlation plots. (**b**) Bland–Altman plots.

The number of subjects available for multivariate linear regression analysis after the outlier removal was ≥14 for all the CV parameters associated with the armband BCG except SV (12 subjects). Multivariate linear regression analysis suggested that each of the CV parameters of interest may be accurately estimated by a combination of as few as two characteristic features. In contrast, the best correlation coefficients achieved by univariate characteristic features were in general low (<0.57) for all CV parameters. For the armband BCG at the univariate level, DP was correlated with PTT_J_ (r = −0.36 ± 0.12) and PTT_I_ (r = −0.34 ± 0.15), PP was correlated with PTT_J_ (r = −0.53 ± 0.06) and PTT_I_ (r = −0.48 ±.08), and SP was correlated with PTT_J_ (r = −0.42 ± 0.11). SV, CO, and TPR were most strongly correlated with T_JJ_ (r = 0.34 ± 0.10, −0.57 ± 0.10, and 0.50 ± 0.10). Table 3 shows the best-performing univariate and bivariate regression models associated with the synthetic weighing scale BCG. Figure 5a shows the correlation plot and Figure 5b the Bland–Altman plot between measured versus regressed CV parameters associated with the synthetic weighing scale BCG.

## 4. Discussion

Direct measurement of the CV parameters necessitates inconvenient and costly equipment and procedures as well as trained operators. The BCG is closely associated with the aortic BP. Considering the prior success with the pulse contour techniques in deriving the CV parameters from arterial BP waveforms, the BCG may have potential value in estimating the CV parameters. Yet, prior work to investigate the feasibility of estimating the CV parameters from the BCG is quite rare. This work rigorously examined, perhaps for the first time, the relationship between the characteristic features in the limb BCG and the CV parameters.

### 4.1. Potential of Scale and Armband BCG in CV Parameter Estimation

The results from the regression analysis suggest that the limb BCG may have the potential to enable unobtrusive CV parameter estimation. For the weighing scale BCG, the pair of two features could achieve close correlations with CV parameters (r ≥ 0.85 for all BP and r ≥ 0.73 for SV, CO, and TPR on the average; Table 3). For the armband BCG, the pair of two features extracted from the synthetic weighing scale BCG transformed from the armband BCG could likewise achieve close correlations with CV parameters (r ≥ 0.73 for all BP, r ≥ 0.75 for CO and TPR, and r = 0.64 for SV on the average; Table 3). In general, the weighing scale BCG outperformed the armband BCG. This may be attributed to (i) the more stable measurement setting for the weighing scale BCG relative to the armband BCG and (ii) the errors induced by the transformation of the armband BCG to the synthetic weighing scale BCG (see Section 4.4 for details). Indeed, the upper limb may be more susceptible to involuntary movement than the lower limb in contact with the weighing scale. Furthermore, the synthetic weighing scale BCG transformed from the armband BCG is not exactly identical to the weighing scale BCG (which may also explain why the features selected for weighing scale BCG and synthetic weighing scale BCG were not identical in Table 3). Combined, these artifacts may result in the deterioration in efficacy of the armband BCG relative to the weighing scale BCG in estimating the CV parameters. Regardless, the degree of correlation between the armband BCG and the CV parameters was still adequate.

The adequate correlation between the armband BCG and the CV parameters appears to have benefited from the signal processing procedure developed in this work to transform the armband BCG to weighing scale BCG. Considering the distinct waveform morphology associated with the weighing scale BCG versus the armband BCG, the efficacy of the signal processing procedure may have a significant implication on the feasibility of standardized analysis of both the BCG. Arguably, the improvement in the correlation between the measured versus synthetic weighing scale BCG compared to the correlation between the measured weighing scale versus armband BCG may suggest that the armband BCG may now be analyzed in the same way as the weighing scale BCG, the analysis method for which is much more established in the sense that the weighing scale BCG may approximately represent the whole-body BCG (i.e., the BCG associated with the movement of the main trunk) [16,42]. 

### 4.2. Physiological Relevance of Weighing Scale BCG Features

**Figure 6:**
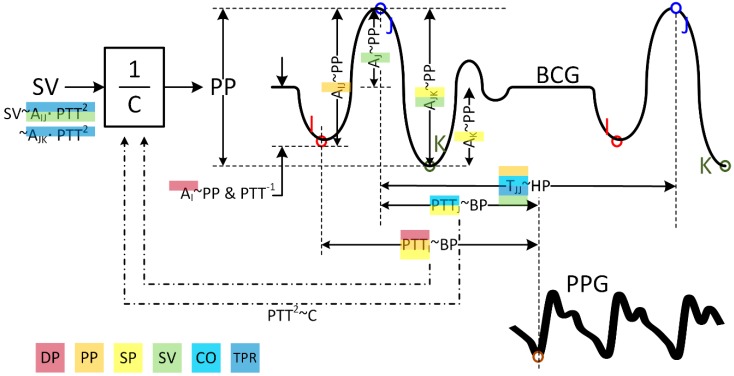
Relationship between the characteristic features in the weighing scale and armband BCG and the CV parameters. BP: blood pressure. DP: diastolic BP. PP: pulse BP. SP: systolic BP. SV: stroke volume. CO: cardiac output. TPR: total peripheral resistance. HP: heart period. C: arterial compliance. “X~Y” means that X and Y are proportional.

The characteristic features in the weighing scale BCG exhibiting close correlation with the CV parameters were physiologically relevant as described below (Table 3 and Figure 6).

First, physiologically relevant weighing scale BCG features exhibited close correlation to the CV parameters in the univariate regression analysis. The correlation of DP with PTT_I_ and PTT_J_ is consistent with the established fact that DP is correlated closely to PTT [49]. The correlation of PP with PTT_I_ and PTT_J_ may be understood by the fact that PP may be (at least in a local sense) inversely proportional to PTT [50,51,52,53]. The correlation of PP with A_JK_ and A_IJ_ may be understood by the fact that the amplitude features A_J_ and A_JK_ may be the surrogates of ascending aortic and descending aortic PP [16] as well as the fact that an increase in PP may lead to an increase in the overall BCG amplitude. The correlation of SP with PTT_I_ and PTT_J_ may be understood from the correlation between DP and PTTs in conjunction with the fact that the hemodynamic interventions considered in this work elicited concurrent increases in both DP and SP. Likewise, physiologically relevant weighing scale BCG features were properly correlated with SV, CO, and TPR in the univariate regression analysis, though not as strong as BP. The correlation of SV with A_J_ is reasonable in that A_J_ may be the surrogate of ascending aortic PP and that SV and PP are proportional to each other if the arterial compliance (AC) does not change largely (PP = SV/AC) [29]. The correlation of CO with T_JJ_, and to a lesser extent, with PTT_I_ and PTT_J_ may also be reasonable by noting that CO is the product of SV and heart rate, T_JJ_ is a surrogate of heart rate, and PTT_I_ and PTT_J_ are correlated with PP (which is proportional to SV). The negative correlation of TPR and PTT_I_ and PTT_J_ appears reasonable given that the changes in BP and TPR are in phase (Figure 3). In contrast, the positive correlation of TPR with T_JJ_ is counter-intuitive in that BP, heart rate, and TPR mostly change in the same direction, except in BH (the change in heart rate may be deduced from SV and CO in Figure 3 as CO/SV). It is speculated that the large inverse change in TPR and heart rate in BH appears to dominate the relatively small in-phase changes in the remaining hemodynamic interventions and, thereby, yielded the positive correlation between TPR and T_JJ_. Hence, the positive correlation between TPR and T_JJ_ as observed in this work may not generalize.

Second, the weighing scale BCG features selected in the bivariate regression analysis were also quite physiologically relevant (Figure 6). DP was regressed with PTT_I_ and A_I_ (r = 0.85 ± 0.02), which is relevant in that A_I_ may be inversely proportional to BP since a decrease in PTT (corresponding to an increase in BP) may be associated with a decrease in A_I_ [16,42]. PP was regressed with PTT_I_ and A_IJ_ (r = 0.85 ± 0.02) consistently to the univariate regression analysis. SP was regressed with PTT_I_ and A_JK_ (r = 0.86 ± 0.02), which is relevant in that A_JK_ may represent PP as mentioned above [16]. SV was regressed with A_J_ and A_JK_ (r = 0.73 ± 0.04), which is supported by the close relationship between these amplitude features and PP [16] and the proportionality between PP and SV under small AC change [29]. SV was also regressed well with T_JJ_ and RMS (r = 0.73 ± 0.04), which may be due to the inversely proportional change between SV and HR (Figure 3; which may be specific to the data analyzed in our work due to large changes in HR and thus may not generalize) and the proportional association between the amplitude features and RMS. CO was regressed with T_JJ_ (which is consistent with the univariate regression case) and PTT_J_ (r = 0.76 ± 0.05). The correlation between CO and PTT_J_ appears relevant because CO and SV are proportional, SV and PP may be proportional, and PP is locally inversely proportional to PTT (as stated above) [50,51,52,53]. TPR was regressed with T_JJ_ (consistent with the univariate regression analysis) and A_IJ_·PTT_I_^2^ (r = 0.77 ± 0.03). Considering that A_IJ_ may serve as a surrogate of PP (as stated above) and that PTT^2^ is proportional to AC according to the wave speed equation [49], A_IJ_·PTT_I_^2^ may be regarded as a surrogate of SV. Hence, it may qualify for a feature to track the trend of TPR given its inversely proportional relationship to TPR (r = −0.32 ± 0.08; Figure 3).

### 4.3. Physiological Relevance of Armband BCG Features

The characteristic features in the synthetic weighing scale BCG transformed from the armband BCG exhibiting close correlation with the CV parameters were physiologically relevant to a large extent as described below (Table 3 and Figure 6).

First, many physiologically relevant synthetic weighing scale BCG features exhibited correlation to the CV parameters in the univariate regression analysis consistently to the weighing scale BCG. However, the degree of correlation was not as strong as the weighing scale BCG.

Second, the synthetic weighing scale BCG features selected in the bivariate regression analysis were likewise quite physiologically relevant and largely consistent with the weighing scale BCG (Figure 6). DP was regressed with PTT_I_ and A_I_ (r = 0.73 ± 0.04). PP was regressed with PTT_I_ and T_JJ_ (r = 0.74 ± 0.04). PTT_I_ may have been selected since it changed in the opposite direction to DP, PP, and SP in this work. T_JJ_ may have been selected since it exhibited a positive correlation with SV in this work (which may be deduced from SV and CO in Figure 3). SP was best regressed with A_JK_ and A_IJ_·PTT_I_^2^ (r = 0.73 ± 0.04). This correlation may be understood in that SP and PP mostly changed in the same direction in response to the interventions considered in this work (Figure 3). However, SP was also well regressed with the pair of PTT_J_ and an amplitude feature (e.g., PTT_J_-A_K_: r = 0.72 ± 0.05). These correlations may be readily interpreted in that PTT and amplitude features may represent DP and PP, respectively [1,16]. SV was regressed with T_JJ_ and A_IJ_·PTT_I_^2^ (r = 0.64 ± 0.06). T_JJ_ may have been selected since it exhibited a positive correlation with SV in this work as stated above, while A_IJ_·PTT_I_^2^ may be a meaningful surrogate of SV as stated earlier. CO was regressed with T_JJ_ and PTT_J_ (r = 0.76 ± 0.04), which may be relevant in that T_JJ_ and PTT_J_ may represent heart rate and PP (which in general correlates with SV; also DP and PP varied in the same direction in response to the hemodynamic interventions considered in this work as shown in Figure 3), respectively. TPR was regressed with T_JJ_ and A_JK_·PTT_I_^2^ (r = 0.75 ± 0.05) similarly to the weighing scale BCG.

### 4.4. Summarizing Remarks and Study Limitations

In summary, the results obtained from this work provide several important implications. First, the characteristic features in the limb BCG have the potential for unobtrusive estimation of CV parameters. Indeed, for both the weighing scale and armband BCG, the pair of as few as two features could achieve close correlation with the CV parameters. Second, the characteristic features selected by the multivariate regression analysis appeared to be largely interpretable (meaning that the selected characteristic features were to a large extent congruent with the physiological insights [16,42]). Indeed, despite the fact that the multivariate regression analysis conducted in this work was predominantly a data-mining exercise, the majority of the characteristic features selected by the analysis were physiologically relevant and consistent with the findings derived from our prior mathematical model-based analysis of the BCG [16,42] (see Section 4.2 and Section 4.3 for details). Hence, the BCG features identified to exhibit close association with CV parameters in this work may be generalizable to other independent datasets. Third, PTT may make significant contributions in CV parameter estimation. Indeed, PTT was selected in all the bivariate regression analyses derived for BP (DP, PP, and SP) in this work. In comparison with our prior work that investigated the association between the characteristic features in the wrist BCG and BP (r = 0.75 for both DP and SP on the average when three predictors were employed) [14], this work achieved much higher correlation with less number of predictors (i.e., two) by including PTT. From this standpoint, it may be of interest to see the potential value of pulse arrival time (PAT) in further improving the association between the limb BCG and CV parameters. In fact, existing work suggests that PAT may serve as a good characteristic feature for SP [49,50,54] as well as CV parameters via the pre-ejection period (which has implications on the heart contractility). One practical consideration may be that the use of PAT necessitates the measurement of the ECG, which generally requires conventional electrodes or two-handed user maneuvers [55]. In this regard, an accuracy–convenience trade-off may need to be made.

This study has a few limitations. First, the signal processing procedure for transforming the armband BCG to the synthetic weighing scale BCG was empiric. The application of two integrations to the armband BCG to yield the armband displacement can be well justified in that the armband displacement may be identical to the weighing scale BCG (which is essentially a displacement measurement) if the body is assumed to be perfectly rigid. However, our choice of the cut-off frequency for the post-integration high-pass filtering of the synthetic weighing scale BCG may not be optimal: although the cut-off frequency was chosen consistently using a set procedure (Figure 2), the procedure was primarily based on empiric attempts to make the power spectra associated with the weighing scale BCG and the synthetic weighing scale BCG look comparable in a qualitative sense (i.e., the amplitudes and locations of the spectral peaks). In future work, this weakness needs to be investigated, so that more effective and robust signal processing procedure for transforming the armband BCG to the weighing scale BCG can be conceived and developed. One possibility may be to explicitly account for the body compliance in the signal processing procedure, by incorporating into it a mathematical model of the body biomechanics that can predict the alteration of the limb BCG waveform due to the compliance and elasticity of the tissues and joints. Second, the study participants were quite homogenous in terms of age and CV health. It is important to investigate if the findings from this work remain valid in a wider group of subjects (e.g., subjects with pacemakers [56] and cardiovascular disease [57]). Third, the CV parameters were measured using a non-invasive device (ccNexfin). Despite the widespread use of the device used in this study in research and its demonstrated accuracy [26], it is possible that the reference CV parameters were associated with inaccuracies due to, e.g., group-average formula used in its pulse contour algorithm [26]. Fourth, this work only investigated the feasibility of estimating the trend of CV parameters. In addition, the regression analysis was conducted in a subject-specific setting. To leverage the results of this work practically in real-world conditions, the characteristic features in the BCG must be calibrated to CV parameters. Hence, future work must investigate subject-specific calibration of the characteristic features in the BCG to CV parameters.

## 5. Conclusions

In this work, we demonstrated that (i) the characteristic features in the limb BCG exhibit close correlation with the CV parameters, and (ii) the characteristic features representative of the CV parameters are largely relevant from a physiological standpoint. Future work must be conducted to translate the findings of this work to more realistic CV parameter estimation techniques.

## Figures and Tables

**Table 1:** Characteristic features extracted from the ballistocardiogram (BCG) in conjunction with the photoplethysmogram (PPG).

Symbol	Definition
PTT_I_	Time interval between BCG I wave and PPG foot
PTT_J_	Time interval between BCG J wave and PPG foot
PTT_K_	Time interval between BCG K wave and PPG foot
T_IJ_	Time interval between BCG I wave and J wave
T_JK_	Time interval between BCG J wave and K wave
T_IK_	Time interval between BCG I wave and K wave
T_JJ_	Time interval between J waves of two consecutive BCG beats
A_I_	Amplitude of BCG I wave
A_J_	Amplitude of BCG J wave
A_K_	Amplitude of BCG K wave
A_IJ_	Amplitude difference between I wave and J wave
A_JK_	Amplitude difference between J wave and K wave
A_IJ_·PTT_I_^2^	Surrogate of SV^*^
A_JK_·PTT_I_^2^	Surrogate of SV^*^
RMS	Root mean square of BCG waveform s[i], i = 1~n: $\overset{n}{\underset{i=1}{\sum }}\sqrt{s{\left[i\right]}^{2}/n\;}$
E	Energy of BCG waveform s[i], i = 1~n: $\overset{n}{\underset{i=1}{\sum }}s{\left[i\right]}^{2}$

*: Considering that A_IJ_ and A_JK_ are approximately associated with PP and PTT^2^ is proportional to arterial compliance, A_IJ_·PTT_I_^2^ and A_JK_·PTT_I_^2^ are approximately associated with stroke volume (SV).

**Table 2:** Extremum regions of cardiovascular (CV) parameters in individual rest and intervention periods.

	R1	CP	R2	MA	R3	SB	R4	BH	R5
DP	Min	Max	Min	Max	Min	Min	Min	Max	Min
PP	Min	Max	Min	Max	Min	Min	Min	Max	Min
SP	Min	Max	Min	Max	Min	Min	Min	Max	Min
SV	Max	Min	Max	Min	Max	Min	Max	Min	Max
CO	Min	Max	Min	Max	Min	Min	Min	Min	Min
TPR	Min	Max	Min	Max	Min	Min	Min	Max	Min

**Table 3:** Representative univariate and bivariate regression models associated with weighing scale ballistocardiogram (BCG) and synthetic weighing scale BCG transformed from armband BCG.

	DP	PP	SP	SV	CO	TPR
Weighing Scale BCG: Univriate (r: mean ± SE)						
Features	PTT_I_	PTT_I_	PTT_I_	A_J_	T_JJ_	PTT_I_
r	0.81 ± 0.02	0.65 ± 0.05	0.82 ± 0.02	0.50 ± 0.09	0.57 ± 0.11	0.58 ± 0.07
Synthetic Weighing Scale BCG: Univariate (r: mean ± SE)						
Features	PTT_J_	PTT_J_	PTT_J_	T_JJ_	T_JJ_	T_JJ_
r	0.36 ± 0.12	0.53 ± 0.06	0.42 ± 0.11	0.34 ± 0.10	0.57 ± 0.10	0.50 ± 0.10
Weighing Scale BCG: Bivariate (r: mean ± SE)						
Features	PTT_I_, A_I_	PTT_I_, A_IJ_	PTT_I_, A_JK_	A_J_, A_JK_	T_JJ_, PTT_J_	T_JJ_, A_IJ_·PTT_I_^2^
r	0.85 ± 0.02	0.85 ± 0.02	0.86 ± 0.02	0.73 ± 0.04	0.76 ± 0.05	0.77 ± 0.03
Synthetic Weighing Scale BCG: Bivariate (r: mean ± SE)						
Features	PTT_I_, A_I_	T_JJ_, PTT_I_	A_JK_, A_IJ_·PTT_I_^2^	T_JJ_, A_IJ_·PTT_I_^2^	T_JJ_, PTT_J_	T_JJ_, A_JK_·PTT_I_^2^
r	0.73 ± 0.04	0.74 ± 0.04	0.73 ± 0.04	0.64 ± 0.06	0.76 ± 0.04	0.75 ± 0.05
